# Metal–organic framework derived single-atom catalysts for electrochemical CO_2_ reduction†

**DOI:** 10.1039/d2ra06302f

**Published:** 2022-11-14

**Authors:** Mengna Xie, Jiawei Wang, Xian-Long Du, Na Gao, Tao Liu, Zhi Li, GuoPing Xiao, Tao Li, Jian-Qiang Wang

**Affiliations:** Key Laboratory of Interfacial Physics and Technology, Shanghai Institute of Applied Physics, Chinese Academy of Sciences Shanghai 201800 China duxianlong@sinap.ac.cn; Engineering Research Center of Large-Scale Reactor Engineering and Technology, Ministry of Education, State Key Laboratory of Chemical Engineering, East China University of Science and Technology Shanghai 200237 China; University of Chinese Academy of Sciences Beijing 100049 China; Dalian National Laboratory for Clean Energy, Chinese Academy of Sciences Dalian 116023 China; Shandong Energy Group Co., Ltd. Jinan 250014 China

## Abstract

With maximum atomic utilization, transition metal single atom catalysts (SACs) show great potential in electrochemical reduction of CO_2_ to CO. Herein, by a facile pyrolysis of zeolitic imidazolate frameworks (ZIFs) assembled with tiny amounts of metal ions, a series of metal–nitrogen–carbon (M–N–C) based SACs (M = Fe, Ni, Mn, Co and Cu), with metal single atoms decorated on a nitrogen-doped carbon support, have been precisely constructed. X-ray photoelectron spectroscopy (XPS) for M–N–C showed that the N 1s spectrum was deconvoluted into five peaks for pyridinic (∼398.3 eV), M–N coordination (∼399.6 eV), pyrrolic (∼400.4 eV), quaternary (∼401.2 eV) and oxidized (∼402.9 eV) N species, demonstrating the existence of M–N bonding. High-angle annular dark-field scanning transmission electron microscopy (HAADF-STEM) indicates homogeneous distribution of metal species throughout the N-doped carbon matrix. Among the catalysts examined, the Fe–N–C catalyst exhibits the best catalytic performance in electrocatalytic CO_2_ reduction reaction (CO_2_RR) with nearly 100% faradaic efficiency for CO (FE_CO_) at −0.9 V *vs.* the reversible hydrogen electrode (RHE). Ni–N–C is the second most active catalyst towards CO_2_RR performance, then followed by Mn–N–C, Co–N–C and Cu–N–C. Considering the optimum activity of Fe–N–C catalyst for the CO_2_RR, we then further investigate the effect of pyrolysis temperature on CO_2_RR of the Fe–N–C catalyst. We find the Fe–N–C catalyst pyrolyzed at 1000 °C exhibits the best catalytic activity in CO_2_RR with excellent CO selectivity.

## Abstract 2

A series of well-defined M–N–C based SACs (M = Fe, Ni, Co, Mn and Cu) was synthesized *via* a facile pyrolysis of metal-doped ZIF-8 and applied in electrocatalytic CO_2_ reduction to CO. Fe–N–C based SACs possess highest activity for CO_2_RR.

## Graphical abstract

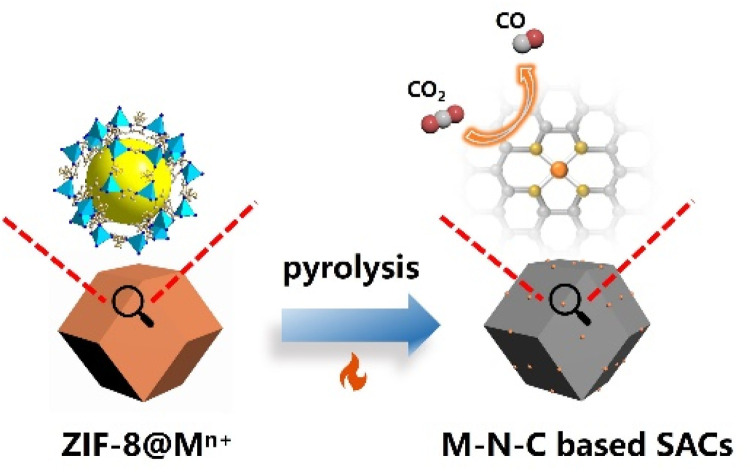

## Introduction

As the main greenhouse gas, massive emission of carbon dioxide (CO_2_) will bring a series of climate change issues^1–4^ (global warming, glacier melting, sea level rise and *etc.*), posing a serious threat to the survival and development of mankind and the biological community.^5,6^ Among the multitudinous approaches for utilization and conversion of CO_2_,^7^ electrochemical CO_2_ reduction reaction (CO_2_RR) is considered as a promising strategy to reduce the accumulation of CO_2_ in the atmosphere^5,8^ and obtain high value-added products, achieving carbon circulation *via* renewable energy sources.^9–11^ Of the reaction pathways possible, CO_2_ conversion into CO has attracted much attention for its usage in the production of a wide range of industrial chemicals such as methane,^12^ ethylene,^13^*n*-propanol^14^ and so on.^15^

Single-atom catalysts (SACs) of transition metals are an excellent candidate due to their remarkable catalytic performances and maximized atom utilization.^16–21^ Benefiting from the well-defined active centers,^22^ the monodisperse metal atoms in SACs have higher coordination unsaturation and more homogeneous structure.^23^ Moreover, SACs possess optimal metal utilization by the exposure of active sites in the catalytic process.^24^ In particular, single metal decorated N-doped carbon (M–N–C) materials have attracted growing attention as alternatives to noble metal catalysts^25,26^ to be applied in CO_2_RR to converse CO_2_ into CO due to the benefits of low cost, abundance, and good catalytic capability at low overpotentials.^27–30^ Metal–organic frameworks (MOFs) are a kind of hybrid materials composed of organic ligands and metal ions or clusters,^31^ attracting wide attention due to the diversity of their structure and tunable physicochemical properties.^32,33^ In addition, MOF synthesized from nitrogen-containing organic compounds like zeolitic imidazolate frameworks (ZIFs) is rich in nitrogen and carbon, which is an ideal precursor for the preparation of porous nitrogen doped materials.^34^ N-doped carbon materials are a class of porous, rich nitrogen-based carbon supports to stabilize single atoms by furnishing enormous opportunity to constitute coordination environment.^35,36^ To obtain M–N–C based SACs, pyrolysis is an essential procedure and as a result, a strong metal–nitrogen coordination bond is formed by metal centres and the abundant nitrogen source of metal–organic frameworks (MOFs).^37^ In this way, Ni *et al.*^25^ synthetized Fe–N–C based SACs by pyrolysis of carbon nitride in the presence of only tiny amounts of Fe salt, which are demonstrated high effective for CO_2_-to-CO conversion even in concentrated electrolyte. However, N-doped carbon substrate always display various characteristics, resulting in the different activity of M–N–C based SACs even with the same metal centre.

We previously reported that a well-defined Fe-based single-atom catalyst for electrocatalytic CO_2_ reduction to CO with highly efficient selectivity and catalytic activity was synthesized *via* a facile pyrolysis of Fe-doped zinc (Zn) 2-methylimidazolate framework (ZIF-8). Dominantly, Fe single-atomic sites exhibit optimum activity in producing CO, presenting a current density of 46.5 mA cm^−2^, with nearly 100% FE for CO (at −0.9 V *vs.* RHE). We correlate the size of Fe NPs with their CO_2_RR performance and demonstrate that further increase in Fe NP size leads to a visible decrease in CO_2_RR selectivity. We herein extend the previous work, and focus on the role of the metal center of the M–N–C catalysts and synthesize a series of M–N–C based SACs (M = Fe, Ni, Co, Mn and Cu) towards selective CO_2_ reduction, starting from ZIF-8 (ref. 38 and 39) that is widely applied as the N–C material substrate in MOFs. Fe–N–C catalyst has the best electrocatalytic performance for CO_2_RR reaction among all M–N–C catalysts, then we combine both electrochemical impedance spectroscopy and CO_2_ adsorption experiments to explore the reason of Fe–N–C for high CO_2_RR activity. Our work has the potential for guiding future rational design of more non-noble SACs with cost efficiency for CO_2_RR.

## Experimental section

### Material

Ferric nitrate nonahydrate (Fe(NO_3_)_3_·9H_2_O), nickel nitrate hexahydrate (Ni(NO_3_)_2_·6H_2_O), manganese nitrate hexahydrate (Mn(NO_3_)_2_·6H_2_O), cobalt nitrate hexahydrate(Co(NO_3_)_2_·6H_2_O), copper nitrate trihydrate (Cu(NO_3_)_2_·3H_2_O), zinc nitrate hexahydrate (Zn(NO_3_)_2_·6H_2_O), 2-methyl imidazole (2-MeIM), potassium bicarbonate (KHCO_3_) and methanol (MeOH) were all purchased from Titan Scientific Co., Ltd. Nafion 117 was supplied by Sigma-Aldrich. All chemicals were analytical grade and used directly without additional treatment.

### Synthesis of ZIF-8

The preparation of ZIF-8 followed previously reported synthetic procedures.^40^ First, Zn(NO_3_)_2_·6H_2_O (0.558 g) and 2-MeIM (0.616 g) were dissolved separately in 15 mL methanol. Then, the two solutions were mixed together under ultrasound for 15 min at room temperature. White suspension obtained by ultrasound was left at room temperature under static for overnight to grow ZIF-8. The precipitate was centrifuged and washed three times with methanol. Finally, the product was dried in vacuum at 60 °C for 12 h.

### Synthesis of M–N–C based SACs

Taking the synthesis of Fe–N–C as an example. Firstly, 100 mg of the as-obtained ZIF-8 powder was dispersed into methanol (15 mL) under ultrasound for 5 min at room temperature. Next, Fe(NO_3_)_3_ aqueous solution (100 mg mL^−1^, 50 μL) was slowly added to the mixed solution dropwise under stirring at room temperature. The mixture was kept under stirring for 24 h so that the ferric salts were absorbed completely. Afterwards, the above mixture was centrifuged and dried in a vacuum oven at 60 °C for 12 h. The samples were transferred into a porcelain boat and heated in a tubular furnace to 1000 °C (heating rate 5 °C min^−1^) for 2 h under Ar atmosphere (40 mL min^−1^). The catalyst was further subjected to acid washing by sonicating it in 10 mL of 1 M nitric acid (HNO_3_, 10 hours) to remove extra-large-sized Fe nanoparticles. Then, the sample was centrifuged and dried in a vacuum oven at 60 °C for 12 h. Finally, Fe–N–C was obtained as black powders. The synthesis of Fe–N–C-*T* was the same to that of Fe–N–C, except that the pyrolysis temperature was adjusted. In this work, *via* changing the pyrolysis temperature to 800 °C, 900 °C, 950 °C, 1000 °C and 1100 °C respectively, five corresponding samples were obtained, which were Fe–N–C-800, Fe–N–C-900, Fe–N–C-950, Fe–N–C-1000 and Fe–N–C-1100.

The Ni–N–C, Mn–N–C, Co–N–C and Cu–N–C were synthesized following the same procedure as Fe–N–C expect for Ni(NO_3_)_2_ aqueous solution(100 mg mL^−1^, 50 μL), Mn(NO_3_)_2_ aqueous solution(100 mg mL^−1^, 50 μL), Co(NO_3_)_2_ aqueous solution(100 mg mL^−1^, 50 μL) and Cu(NO_3_)_2_ aqueous solution(100 mg mL^−1^, 50 μL).

### Synthesis of N–C

ZIF-8 N-doped carbon substrates were prepared through pyrolysis of ZIF-8 powder directly in a tubular furnace with the same heating procedure of Fe–N–C as mentioned above under Ar atmosphere.

### Physicochemical characterization

The crystal structures of the synthesized materials were determined by X-ray diffraction (XRD). The XRD patterns were recorded by using a Germany Bruker D8 advance X-ray diffractometer using nickel filtered Cu Kα (*λ* = 1.54178 Å) radiation with a scanning angle (2*θ*) of 20–80°, a scanning speed of 2° min^−1^, and a voltage and current of 40 kV and 40 mA. X-ray photoelectron spectroscopy (XPS) was acquired on a thermo ESCALAB 250 with Al Kα (*hλ* = 1486.6 eV) as the excitation source. The Fourier transform infrared (FT-IR) spectra of the sample was recorded using a Frontier FT-IR spectrometer (Bruker, Vertex 80) with KBr pellets in the range of 4000–400 cm^−1^. Thermogravimetry and derivative thermogravimetric curve (TG-DTG) were performed on a NETZSCH STA449F3 apparatus, and about 5.5 mg sample was heated at 5 °C min^−1^ up to 1000 °C in Ar. CO_2_ adsorption experiments were performed with a Micromeritics ASAP 2460 analyzer and CO_2_ uptake was calculated based on a Brunauer–Emmett–Teller (BET) method. Scan electron microscopy (SEM) was performed by using a ZEISS Merlin Compact Field Emission Scanning Electron Microscope with an acceleration voltage of 5 kV. Transmission electron microscopy (TEM) and energy-dispersive X-ray spectroscopy (EDS) characterizations were carried out using a Tecani-G2 T20 and F20 operating at an acceleration voltage of 200 kV. The M–N–C was imaged with high-angle annular dark-field scanning transmission electron microscopy (HAADF-STEM). The HAADF-STEM images were imaged by using a scanning/transmission electron microscope operated at 300 kV, equipped with a probe spherical aberration corrector.

### Inductively coupled plasma mass spectroscopy (ICP-MS)

The metal ion concentrations of the samples were determined by the high resolution inductively coupled plasma mass spectroscopy (Attom, Nu Instruments, UK). Taking the examination of Fe–N–C as an example, commercially Fe standard solution (1000 mg L^−1^, Titan) was used for calibration. The calibration solutions of 10, 5, 1 and 0.1 ng mL^−1^ Fe were prepared by diluting the Fe standard solution with 2 v/v% HNO_3_. The correlation coefficient of the calibration curve was better than 0.999. All samples were dissolved in concentrated HNO_3_ and then diluted for further measurements.

### Electrochemical measurements

Electrochemical measurements were carried out in a customized flow-type electrolytic cell with gas diffusion electrodes, separating the cathode from the anode with Nafion 117 proton exchange membrane. The experiment was conducted in CO_2_-saturated 0.5 M KHCO_3_ solution under room temperature and atmospheric pressure with a three electrodes system, Pt foil electrode as the counter electrode and Ag/AgCl electrode filled with a saturated KCl solution as the reference electrode. The working electrode was prepared as following: typically, 10 mg of the as-obtained catalyst was dispersed in the mixed solution of isopropanol (960 μL) and Nafion 117 solution (5%, 40 μL) under ultrasonic treatment for 15 minutes to form a uniform ink solution. 100 μL of ink solution was equably sprayed on the hydrophilic side of carbon fiber paper (1 × 1 cm^2^) with a mass loading of 1 mg cm^−2^ and then it was dried in vacuum. All potentials controlled by the electrochemical workstation were then converted to a reversible hydrogen electrode (RHE), which were calculated as *E vs.* RHE = *E vs.* Ag/AgCl + 0.1989 V + 0.0592 V × pH. The supporting electrolyte in the electrolytic cell was 0.5 M KHCO_3_ solution (saturated with CO_2_) by blowing high-purity CO_2_ into the KHCO_3_ solution at a flow rate of 5 mL min^−1^ for at least 0.5 h, the PH value of which was approximately 7. During the experiment, CO_2_ gas (99.99%) was delivered into the cathode with an average rate of 5 mL min^−1^ measured by a universal gas-flow meter. Electrochemical impedance spectroscopy (EIS) tests were performed at an AC voltage amplitude (5 mV), with frequencies ranging from 100 000 Hz to 0.01 Hz. The gas phase product was sent to gas chromatography (GC) connected with the closed electrochemical flow cell online for *in situ* analysis. Porapark Q and 5 A packed column with thermal conductivity detector (TCD) was used to analyze CO_2_, CO and H_2_. HP-AL/M column with flame ionization detector (FID) was used to analyze hydrocarbons in the gas phase. Liquid products were quantified by high-performance liquid chromatography (HPLC) (Agilent 1260) analysis after the electrolysis was finished. The column used was an Aminex HPX 87-H (Bio-Rad) and diluted sulfuric acid (1 mM) was used as the eluent. The temperature of the column was maintained at 40 °C in a column oven, and the separated compounds were detected with a refractive index detector (RID).

### Faradaic efficiency and turnover frequency calculation

The faradaic efficiency (FE) of H_2_ and CO production was calculated as follow:
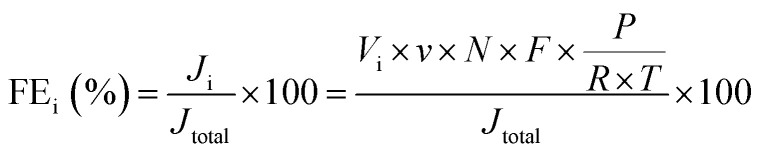
where i represents the product, H_2_ or CO. *J*_i_: partial current density for H_2_ or CO production; *J*_total_: total current density, measured by electrochemical workstation; *V*_i_: the volume concentration of H_2_ or CO, respectively, measured by GC; *v*: average flow rate of CO_2_, which is 5 mL min^−1^; *N*: the number of electrons transferred for product formation, which is 2 for H_2_ or CO; *F*: Faraday constant, 96 485 C mol^−1^; *P*: pressure, Pa; *R*: 8.314 J mol^−1^ K^−1^; *T*: thermodynamic temperature, K.

Turnover frequency (TOF) of H_2_ and CO production was calculated as follow:
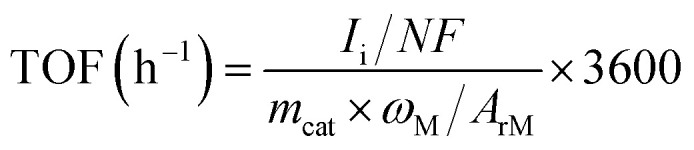
where i represents the product, H_2_ or CO. *I*_i_: partial current for H_2_ or CO production; *N*: the number of electrons transferred for product formation, which is 2 for H_2_ or CO; *F*: Faraday constant, 96 485 C mol^−1^; *m*_cat_: mass of catalyst on working electrode, g; *ω*_M_: mass percentage of single-atom metal in the catalyst; *A*_rM_: atomic mass of single-atom metal.

## Results and discussion

**Fig. 1:**
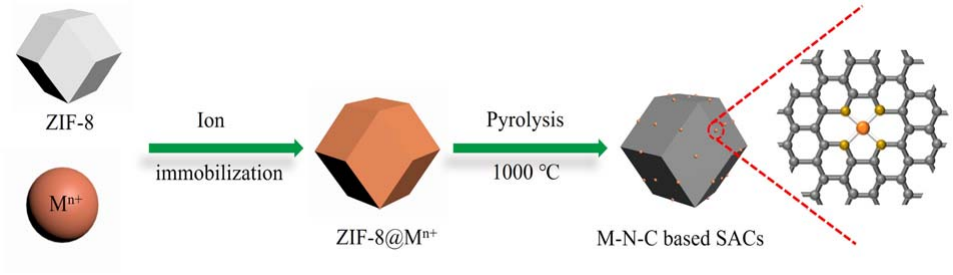
Illustration showing the general fabrication of M–N–C based SACs for electrocatalytic CO_2_ reduction.

**Fig. 2:**
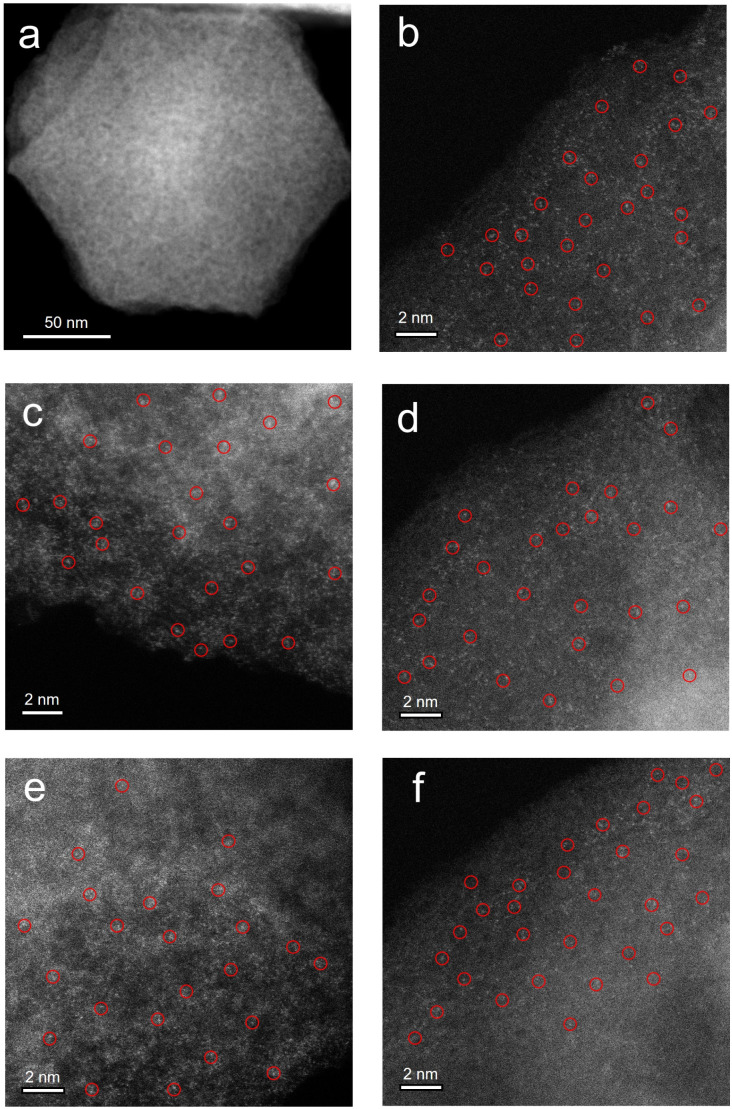
Structural and morphological characterization of M–N–C based SACs (M = Fe, Ni, Mn, Co, Cu). (a) TEM image of Fe–N–C. The aberration-corrected HAADF-STEM images of (b) Fe–N–C, (c) Ni–N–C, (d) Mn–N–C, (e) Co–N–C and (f) Cu–N–C.

**Fig. 3:**
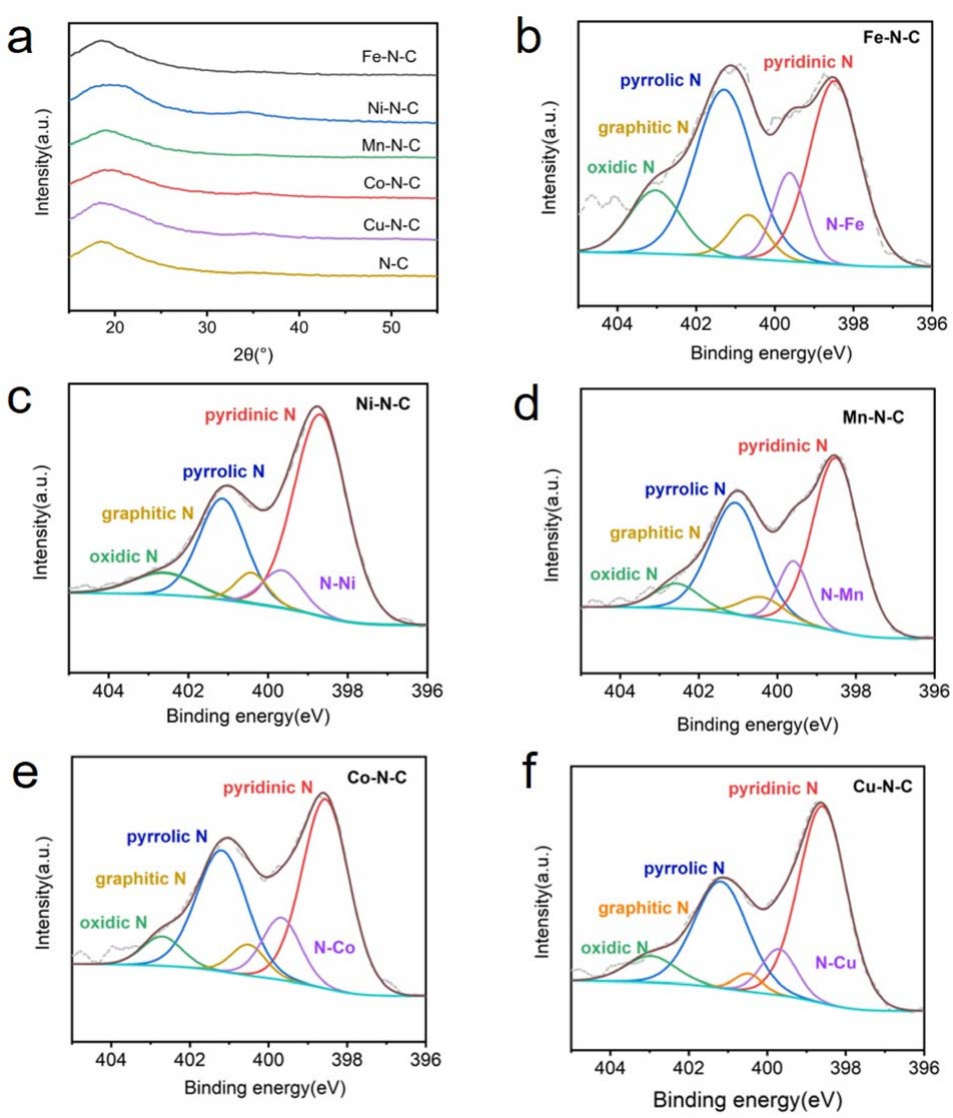
(a) XRD patterns of M–N–C (M = Fe, Ni, Mn, Co, Cu) and metal free N–C. N 1s XPS spectrum of (b) Fe–N–C, (c) Ni–N–C, (d) Mn–N–C, (e) Co–N–C and (f) Cu–N–C.

A series of M–N–C based SACs (M = Fe, Ni, Mn, Co, Cu) have been successfully synthesized by ZIF-8 and metal nitrate *via* a facile pyrolysis strategy followed by the pickling to remove extra-large-sized metal nanoparticles. The synthesis procedure for the catalysts is presented in Fig. 1. Details on preparation of M–N–C (M = Fe, Ni, Mn, Co, Cu) catalysts and ZIF-8 N-doped carbon substrates are provided in experimental section. Furthermore, the effect of calcination temperature on the CO_2_RR performance of Fe–N–C catalyst was explored as Fe–N–C experienced the highest current density and CO faradaic efficiency (FE_CO_) over the other four candidates. Scanning electron microscope (SEM) and transmission electron microscopy (TEM) were carried out to reveal the morphological and structural features of the catalysts. It can be clearly seen from Fig. 2a and S1† that ZIF-8 maintains the uniform rhombic dodecahedral shape with a diameter of about 200 nm before and after high temperature roasting (at 1000 °C under the protection of Ar atmosphere for 2 hours). Therefore, with the general synthetic approach, the obtained M–N–C based SACs involving same nitrogen doped carbon substrate possess uniform rhombic dodecahedral morphology with similar surface area and pore structure, as shown in Fig. S2.† Additionally, no aggregation of metals to either nanoparticles or clusters is observed in SEM or TEM images (Fig. S2 and S3†). The ZIF-8 and ZIF-8 derived carbon and metal catalysts were examined by the X-ray powder diffraction (XRD). As shown in Fig. S4,† the as-prepared sample showed the typical crystal pattern of ZIF-8. Meanwhile, M–N–C showed a broad shoulder peak, which was the same as that of C–N, deriving from pure ZIF-8 (Fig. 3a). This peak was assigned to the (002) plane of the graphitic carbon. No peaks of metallic phase and zinc related peak can be found in the XRD patterns of all five M–N–C catalysts and ZIF-8 derived carbon, in accordance with SEM and TEM results.

To further investigate the atomic distribution of the metals in the M–N–C catalysts, aberration-corrected high-angle annular dark-field scanning transmission electron microscopy (HAADF-STEM) was performed. Taking Fe–N–C as a representative, the isolated bright spots circled indicates homogeneous distribution of Fe species throughout the N-doped carbon matrix in the corresponding HAADF-STEM images of Fe–N–C, presented in Fig. 2b–f. Moreover, the energy-dispersive X-ray spectroscopy (EDS) mappings (Fig. S5†) also confirm the uniform distribution of Fe atoms, similar to those of Ni-, Mn-, Co- and Cu–N–C (Fig. S6–S9†). Quantitatively, the Fe loading in the Fe–N–C is determined to be 0.48% measured by inductively coupled plasma mass spectrometry (ICP-MS) analysis, which is closed to the other M–N–C (M = Ni, Mn, Co, Cu) catalysts (Table S1†).

The catalyst surface chemical composition and state were investigated by X-ray photoelectron spectroscopy (XPS) (Fig. 3b–f). High-resolution N 1s spectra for Fe, Ni, Mn, Co and Cu–N–C show that the N 1s spectrum was deconvoluted into five peaks at pyridinic (∼398.3 eV), N–M moieties (∼399.6 eV), pyrrolic (∼400.4 eV), quaternary (∼401.2 eV) and oxidized (∼402.9 eV) N species,^41,42^ demonstrating the existence of M–N bonding. Notably, pyridinic N predominates the atomic concentration in all five catalysts. The high-resolution Fe 2p spectra (Fig. S10a†) shows that the dominated peak is centered at 711.6 eV, suggesting the partially oxidized Fe species in Fe–N–C. In addition, no peaks assigned to metal nanoparticles can be detected. Corresponding results of XPS spectra (Fig. S10b–e†) are also obtained for Ni-, Mn-, Co- and Cu–N–C catalysts, all confirming the formation of M–N species respectively. The internal structure of the Fe–N–C catalyst was also studied by the FT-IR, as shown in Fig. S11.† Three obvious peaks observed in the IR curve were the O–H stretching vibration (∼3440 cm^−1^), C=C stretching vibration (∼1580 cm^−1^), and C–N & C–O stretching vibration (∼1160 cm^−1^). The weak peaks between 1400 cm^−1^ and 1600 cm^−1^ were come from nitrogen-containing functional groups such as pyrrole nitrogen and pyridine nitrogen formed after carbonization at high temperature.^43^

**Fig. 4:**
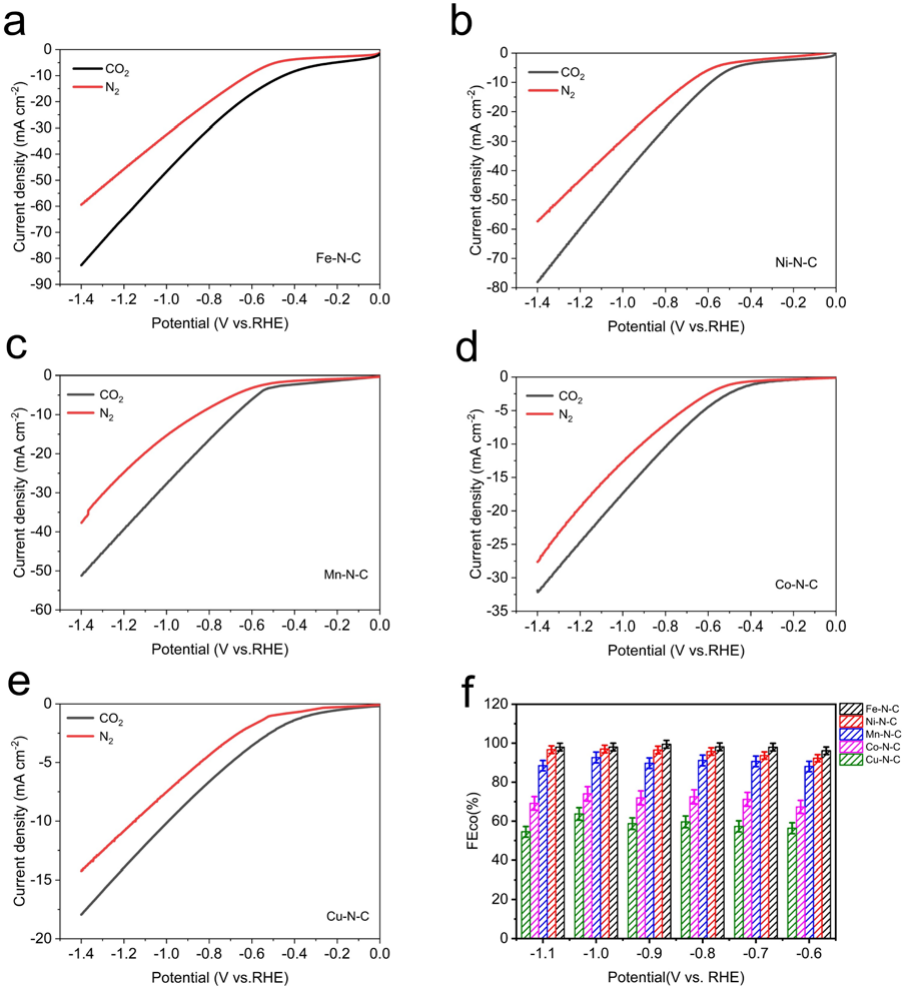
Electrochemical performances for CO_2_ reduction. LSV curves of (a) Fe–N–C, (b) Ni–N–C, (c) Mn–N–C, (d) Co–N–C and (e) Cu–N–C in pure N_2_- and CO_2_-saturated 0.5 M KHCO_3_ at a scan rate of 10 mV s^−1^. (f) The corresponding FE_CO_ at different applied potentials.

To evaluate the catalytic activity and selectivity of M–N–C catalysts for CO_2_-to-CO conversion, the electrocatalytic measurements of the processed catalysts (prepared by drop-casting onto 1 cm × 1 cm carbon paper) were tested in CO_2_-saturated 0.5 M KHCO_3_ electrolyte using a flow cell with a three-electrode configuration at the ambient temperature and pressure. As revealed by linear scanning voltammetry (LSV) measurements (Fig. 4a–e), with the negative shift of potential, all five samples exhibit higher current density under CO_2_-saturated conditions compared to N_2_-saturated conditions, indicating electrochemical reduction of CO_2_ on the M–N–C electrodes.

Clearly, the total current of each electrocatalyst gradually increases with enhancing the reduction potential. Among those catalysts, Fe–N–C and Ni–N–C offer much superior current responses than other M–N–C catalysts, manifesting their excellent catalytic activities towards CO_2_RR. In the meanwhile, Fe–N–C shows a lower cathodic onset potential, suggesting its effective CO_2_ reduction performance. The Nyquist plots show a smaller semicircle diameter of Fe–N–C (Fig. S12†) than other M–N–C catalysts, indicating a faster charge-transfer process for Fe–N–C in CO_2_-saturated 0.5 M KHCO_3_ solution, and finally resulting in enhanced activity for the electrochemical CO_2_ reaction. Besides, we also performed gas adsorption experiments for all M–N–C catalysts in order to better understand their gas capturing capacity, as shown in Fig. S13,† Fe–N–C catalyst has the maximum capacity for CO_2_ capture at atmospheric pressure. This indicates the potential of Fe–N–C catalyst to trap CO_2_ molecules despite the low CO_2_ solubility in the electrolyte. Based on above discussion, we deduced that the Fe–N–C catalyst exhibits the best catalytic performance superiority in electrocatalytic CO_2_ reduction reaction.

**Fig. 5:**
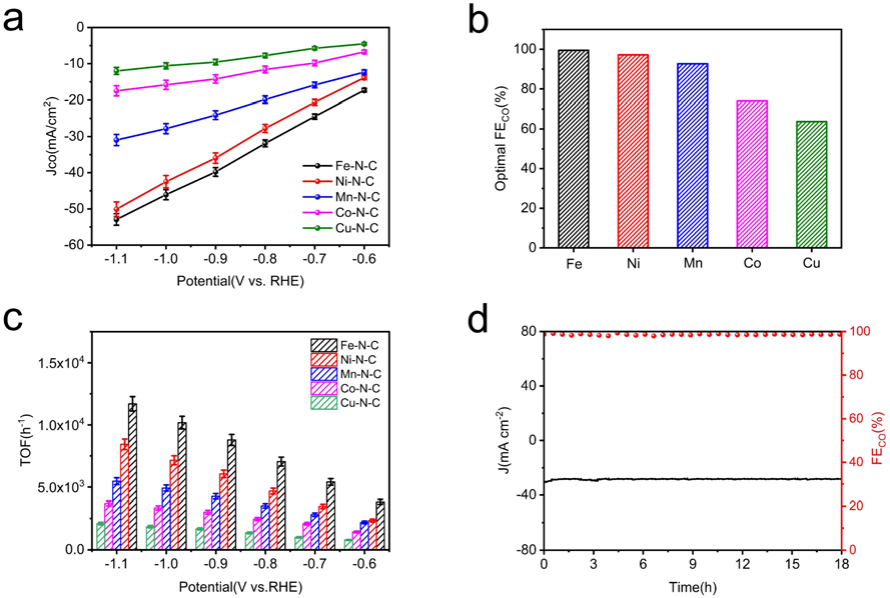
Electrocatalytic CO_2_RR performance of M–N–C catalysts in CO_2_-saturated 0.5 M KHCO_3_ electrolyte within a flow cell. (a) CO partial current density (*J*_CO_). (b) The optimal FE_CO_ and (c) TOFs at different applied potentials of all five catalysts. (d) Stability of Fe–N–C at −0.9 V *vs.* RHE for 18 h continuous electrolysis.

For a more intuitive comparison, the faradaic efficiency of CO (FE_CO_) on the cathode for each catalyst at the different working potentials, varying from −0.6 to −1.1 V *vs.* RHE, has been investigated. No liquid product is detected by high-performance liquid chromatography analysis after electrolysis and the examination results of the gas chromatography show that CO and H_2_ are the main products in all potential ranges. All the data were repeated three times and averaged. It is well-established that H_2_ evolution reaction (HER) is a competing reaction with CO_2_ reduction in CO_2_-saturated electrolytes, therefore the production of H_2_ was also measured during electrolysis. Compared to Co–N–C and Cu–N–C, the three catalysts, Fe–N–C, Ni–N–C and Mn–N–C, display much higher FE_CO_ (Fig. 4f). In particular, Fe–N–C gives the optimum CO selectivity with an ultrahigh FE_CO_ > 99% at −0.9 V *vs.* RHE while Ni–N–C (97% at −1.0 V *vs.* RHE) and Mn–N–C (92% at −1.0 V *vs.* RHE) present inferior FE_CO_ to Fe–N–C during the entire potential range (Fig. 5b). Furthermore, Fe–N–C, with the maximum FE_CO_ among these catalysts, requires a lower potential to selectively reduce CO_2_, which is less energy-consuming than other M–N–C. Meanwhile, the high CO selectivity (over 95%) of Fe– N–C can be maintained at a variety of constant potentials from −0.6 to −1.1 V *vs.* RHE, proving the remarkable selectivity of Fe–N–C for CO_2_RR (Fig. 4f). However, the value of FE_CO_ drops gradually of all catalysts, which could be mainly attributed to the competitive HER at higher applied potentials. For better comparison, the electrochemical performance of CO_2_RR on the metal-free N–C is also measured. According to Fig. S14,† the current density of N–C catalyst is found to be sluggish and its faradaic efficiency for H_2_ production (FE_H_2__) exceeds 80% at all applied potentials, that is, HER dominates in this case. These results demonstrate that the activity and selectivity of catalysts for electrochemical CO_2_ reduction are strongly influenced by the nature of the metal. To have a better understanding, CO partial current densities (*J*_CO_) of all M–N–C catalysts are calculated at the corresponding potential, respectively. It can be seen clearly that Fe–N–C achieves superior *J*_CO_ to other catalysts at any applied potential, which reaches a high value of 52 mA cm^−2^ at −1.1 V *vs.* RHE, provided in Fig. 5a.

A profound understanding of CO_2_RR performance for CO production on M–N–C catalysts was further provided by electrochemical active surface area (ECSA) measurements. As a reference of ECSA, the double layer capacitance (*C*_dl_) is determined according to the scanning rate dependence of cyclic voltammetry (Fig. S15a–e†) by measuring the capacitive current related to it. The ECSA was calculated by *C*_dl_, presented in Fig. S15f,† demonstrating that the Fe–N–C possesses the largest *C*_dl_ (134.5 mF cm^−2^) among the five samples, further supporting the higher catalytic activity due to the higher porosity of Fe–N–C. The *C*_dl_ values decreased sequentially in the order of Ni–N–C, Mn–N–C, Co–N–C and Cu–N–C, which were quantified as 88.2, 83.1, 78.0 and 26.9 mF cm^−2^ respectively, in accordance with *J*_CO_.

For a deeper study, the turnover frequency (TOF) of CO production was used to evaluate the intrinsic activities of M–N–C accordingly. The TOF per active metal site was obtained at different potentials based on their partial CO current densities (Fig. 5c). Obviously, the TOF of Fe–N–C far suppresses that of the other four M–N–C catalysts. A highest TOF of 11 693 h^−1^ was achieved for Fe–N–C at −1.1 V *vs.* RHE and the Ni–N–C is the second most active catalyst with a high TOF of 8437 h^−1^, then followed by TOFs of Mn–N–C, Co–N–C and Cu–N–C, coinciding well with the results of a series of electrochemical tests mentioned. In addition, the best performed Fe–N–C can be continuously operated at −0.9 V *vs.* RHE for 18 h continuous electrolysis with nearly unchanged current density and FE_CO_ at a high value (slightly lower than 100%), unambiguously indicating its remarkable durability for CO_2_RR (Fig. 5d).

Considering the optimum activity of Fe–N–C catalyst for CO_2_RR and the importance of pyrolysis temperature in the synthesis of Fe–N–C to achieve the high performance, a detailed investigation of electrocatalytic activities on pyrolysis products at various temperature was investigated to further optimize the superior CO_2_RR performance of Fe–N–C. Generally speaking, typical pyrolysis temperatures are above 800 °C, at which temperatures ZIF-8 is transformed into a N-doped carbon skeleton and the zinc node with a low boiling point of 907 °C is reduced to the zero valent state and evaporates.^44^ Thermal stability of the as-prepared Fe doped ZIF-8 was predicted using TG-DTG. As shown in Fig. S16,† the weight loss involved two stages: the first one at low temperatures (<150 °C) was ascribed to the evaporation of adsorbed water molecules. And the second one at 350–600 °C was attributed to linker decomposition of ZIF-8, such as CN fragments. When the temperature was elevated to over 900 °C, the weight loss may have resulted from the release of Zn species, leaving the N rich defects.^45^ Thus, we change the pyrolysis temperature from 800 °C to 1100 °C (denoted herein as Fe–N–C-*T*, where *T* are 800 °C, 900 °C, 950 °C, 1000 °C and 1100 °C respectively).

**Fig. 6:**
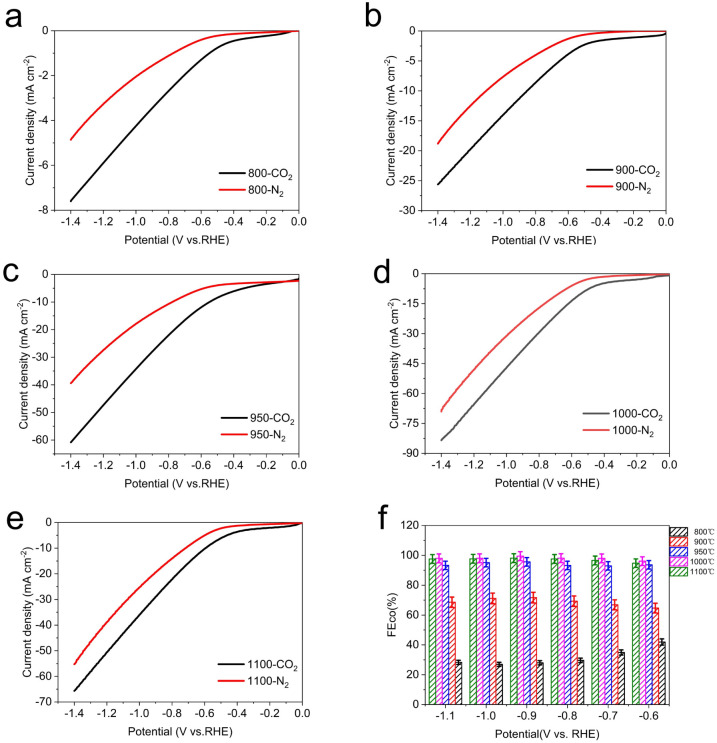
Electrochemical performances for CO_2_ reduction. LSV curves of Fe–N–C-*T* (a) 800 °C, (b) 900 °C, (c) 950 °C, (d) 1000 °C and (e) 1100 °C in pure N_2_- and CO_2_-saturated 0.5 M KHCO_3_ at a scan rate of 10 mV s^−1^. (f) The corresponding FE_CO_ at different applied potentials.

The LSV curve results of Fe–N–C-*T* catalysts show that the total current density for the CO_2_ reduction performance of Fe metal catalysts with different pyrolysis temperature is different (Fig. 6a–e). Especially when the temperature rises to 950 °C, the total current density improves significantly, which is more than twice that at 900 °C under CO_2_-saturated condition. While the total current density drops slightly after the pyrolysis temperature reaches to 1100 °C. Apparently, Fe–N–C-1000 possesses the highest current response whether under CO_2_-saturated or N_2_-saturated atmosphere, compared with those of other Fe–N–C-*T* catalysts.

**Fig. 7:**
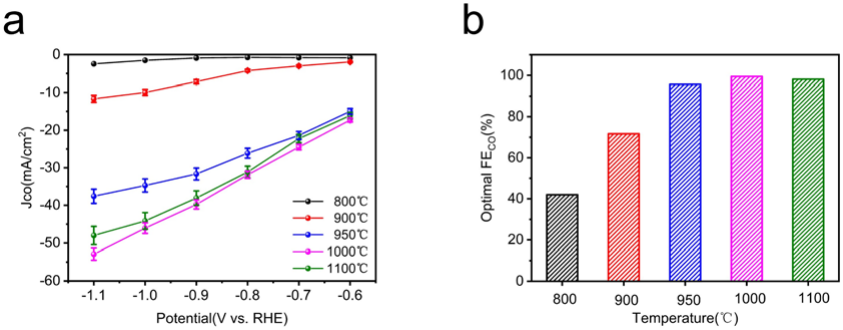
Electrocatalytic CO_2_RR performance of Fe–N–C-*T* catalysts in CO_2_-saturated 0.5 M KHCO_3_ electrolyte within a flow cell. (a) CO partial current density (*J*_CO_); (b) the optimal FE_CO_.

To further understand the effect of pyrolysis temperature of Fe–N–C-*T* on CO_2_ reduction performance, the FE_CO_ of each Fe–N–C-*T* is evaluated. As shown in Fig. 6f, the enhancement can be seen in FE_CO_ from Fe–N–C-800 to Fe–N–C-1000 at any applied potential. Better yet, the selectivity of Fe–N–C-1000 for CO is close to 100% in all potential range, meaning that the competitive HER is dramatically suppressed with FE less than 1%, in sharp contrast to that of Fe–N–C-800. On the basis of the above results, the CO_2_RR performance follows the order Fe–N–C-1000 > Fe–N–C-1100 > Fe–N–C-950 > Fe–N–C-900 > Fe–N–C-800, which justifies FE_CO_, illustrating the high activity of Fe–N–C-1000 (Fig. 7). Therefore, 1000 °C is supposed to be the best pyrolysis temperature among the applied temperatures in this study.

## Conclusions

In summary, a series of well-defined M–N–C based SACs was synthesized *via* a facile pyrolysis of metal-doped ZIF-8 and applied in electrocatalytic CO_2_ reduction to CO. Dominantly, the obtained Fe–N–C catalyst presents highly efficient selectivity and catalytic activity for boosting CO_2_RR performance with nearly 100% FE_CO_ in all applied potential range, compared to other M–N–C catalysts. In addition, Ni–N–C is the second most active catalyst towards CO_2_RR, then followed by Mn–N–C, Co–N–C and Cu–N–C, with corresponding optimal FE_CO_ of 97%, 92%, 74% and 60% respectively, further proved by the results of *J*_CO_ and TOF values. Moreover, we point out the importance of the pyrolysis temperature in the synthesis of Fe–N–C and correlate it with corresponding CO_2_RR performance, ranging from 800 °C to 1100 °C. As a consequence of electrocatalytic activities, Fe–N–C-1000 exhibits superior performance with excellent CO selectivity. Therefore, it can be clearly confirmed that 1000 °C is the optimum pyrolysis temperature among the applied temperatures in this study, in agreement with experimental observations. The family of M–N–C SACs prepared through a facile pyrolysis of zeolitic imidazolate frameworks have the potential for guiding future rational design of more non-noble catalysts for CO_2_RR with cost efficiency.

## Conflicts of interest

The authors declare no competing financial interest.

## Supplementary Material

RA-012-D2RA06302F-s001
