# Supplementary material for: Metal–organic framework derived single-atom catalysts for electrochemical CO2 reduction
Source: RSC Adv. 2022 Nov 14;12(50):32518–25. doi: 10.1039/d2ra06302f (PMC9661489; doi:10.1039/d2ra06302f)
Supplement: RA-012-D2RA06302F-s001 [file RA-012-D2RA06302F-s001.pdf]

Electronic Supplementary Material (ESI) for RSC Advances.
This journal is © The Royal Society of Chemistry 2022

Supporting Information

Metal-organic frameworks derived single-atom catalysts for electrochemical CO_2 reduction

Mengna Xie,‡[ab] Jiawei Wang,‡[ab] Xian-Long Du,*[acd] Na Gao,[ad] Tao Liu,[e] Zhi Li,[e] GuoPing Xiao,[acd] Tao Li[b] and Jian-Qiang Wang[acd]

[a] Key Laboratory of Interfacial Physics and Technology, Shanghai Institute of Applied Physics, Chinese Academy of Sciences, Shanghai 201800

[b] Engineering Research Center of Large-Scale Reactor Engineering and Technology, Ministry of Education, State Key Laboratory of Chemical Engineering, East China University of Science and Technology, Shanghai 200237, China

[c] University of Chinese Academy of Sciences, Beijing 100049, China

[d] Dalian National Laboratory for Clean Energy, Chinese Academy of Sciences, Dalian 116023, China

[e] Shandong Energy Group Co., Ltd., Jinan 250014, China

* Corresponding Author

E-mail: duxianlong@sinap.ac.cn

‡These authors contributed equally to this work.

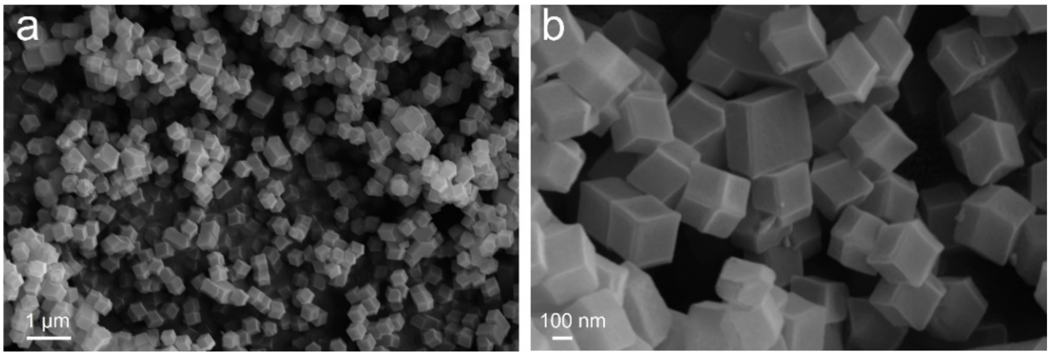

Figure S1. (a) and (b) SEM images of ZIF-8. It is obvious that the synthesized ZIF-8 exhibits a uniform rhomb dodecahedral shape.

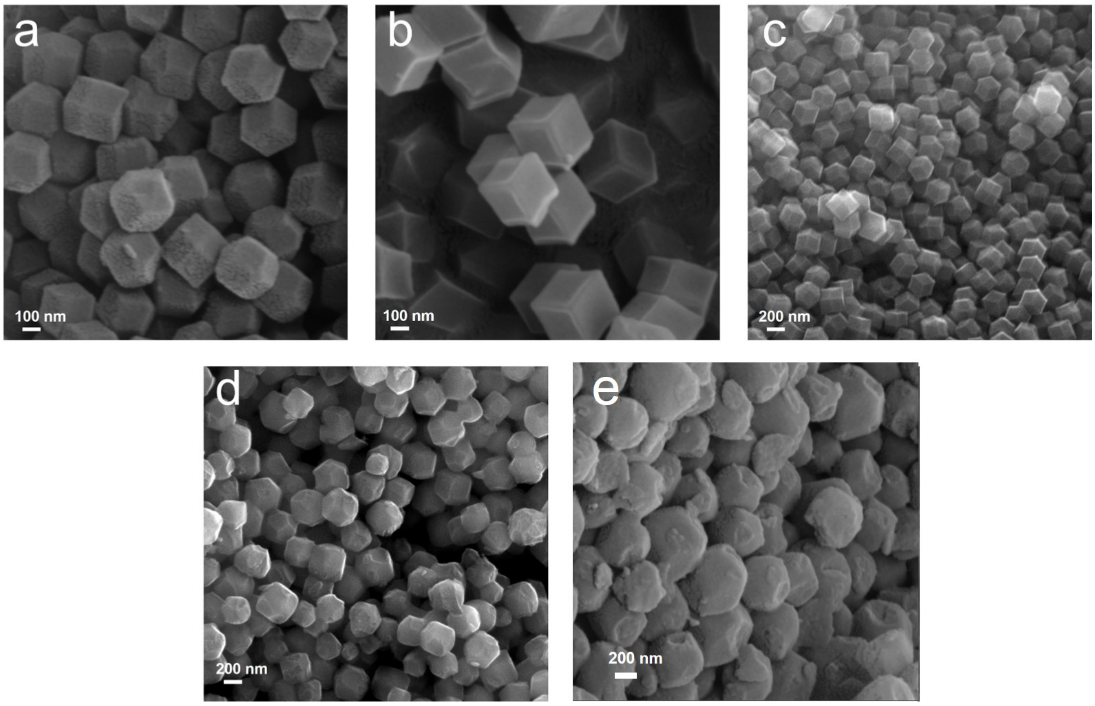

Figure S2. SEM characterization of M-N-C. SEM images of (a) Fe-N-C, (b) Ni-N-C, (c) Mn-N-C, (d) Co-N-C and (e) Cu-N-C.

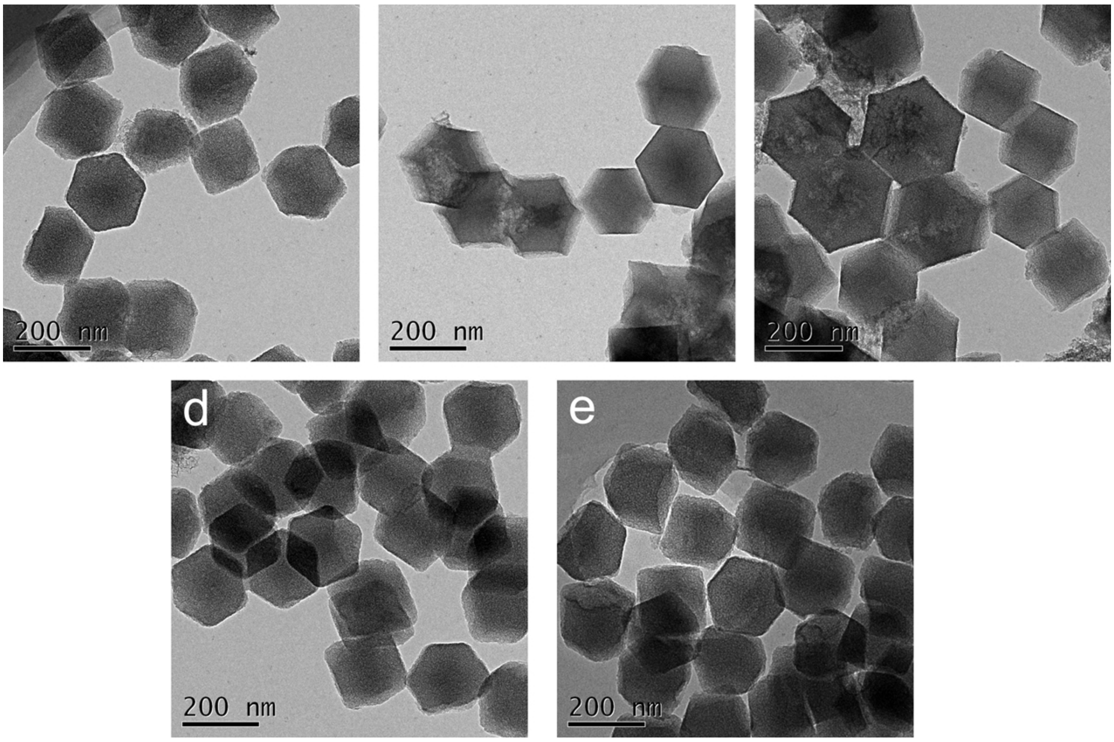

Figure S3. TEM characterization of M-N-C. TEM images of (a) Fe-N-C, (b) Ni-N-C, (c) Mn-N-C, (d) Co-N-C and (e) Cu-N-C.

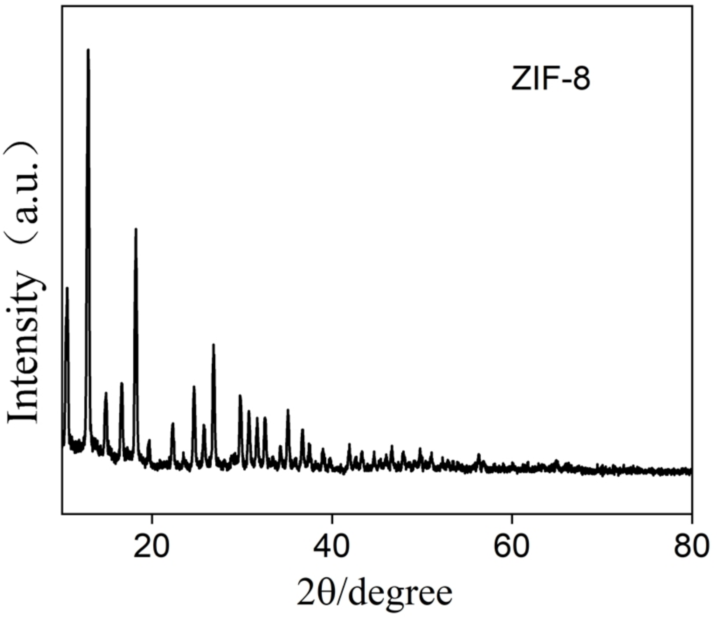

Figure S4. XRD pattern of ZIF-8.

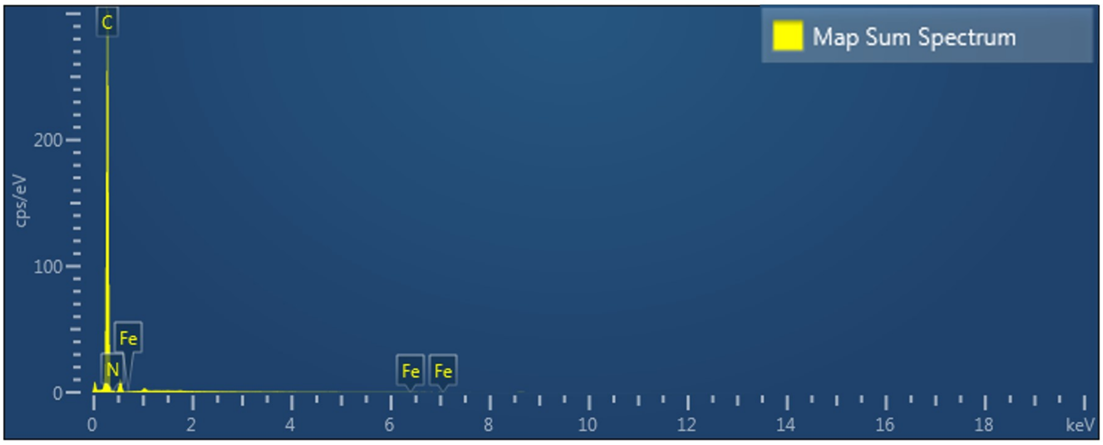

Figure S5. Energy dispersive spectroscopy (EDS) characterization of Fe-N-C. No visible Zn signal locating at 8.63 keV was detected in Fe-N-C, suggesting low residual Zn content.

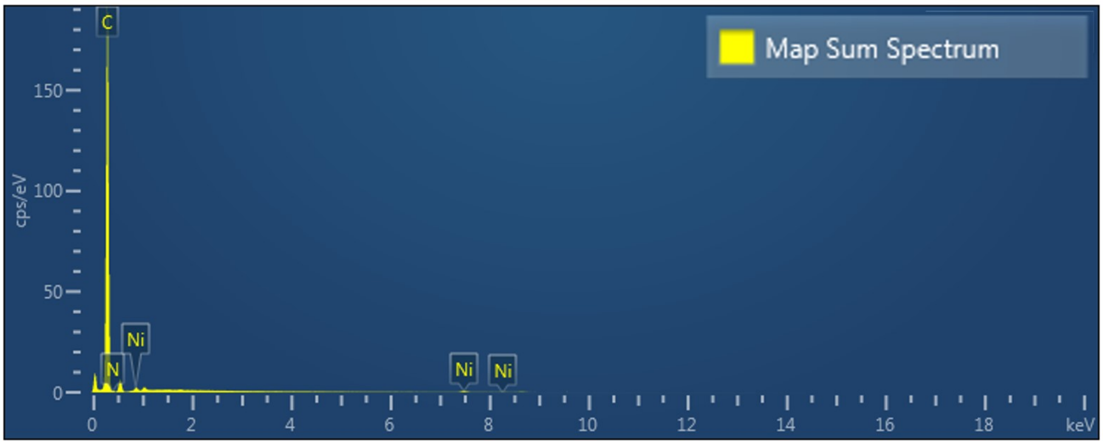

Figure S6. Energy dispersive spectroscopy (EDS) characterization of Ni-N-C. No visible Zn signal locating at 8.63 keV was detected in Ni-N-C, suggesting low residual Zn content.

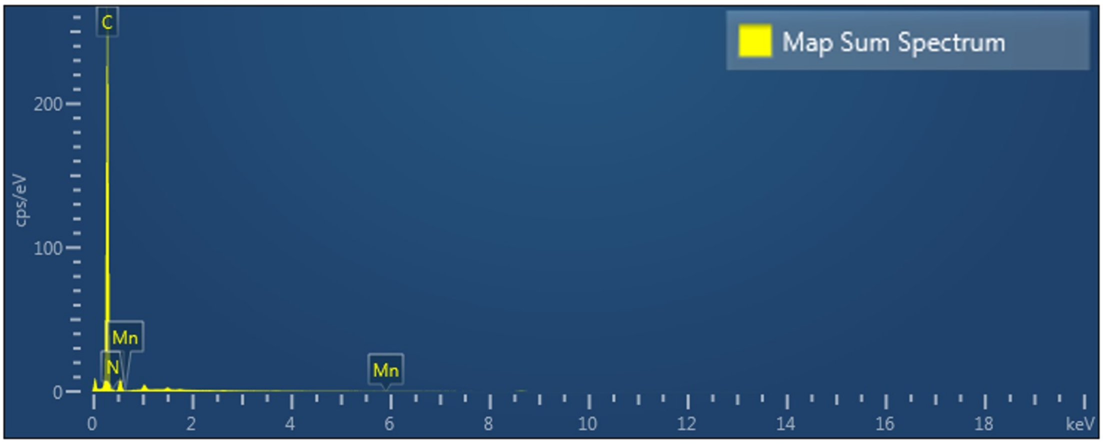

Figure S7. Energy dispersive spectroscopy (EDS) characterization of Mn-N-C. No visible Zn signal locating at 8.63 keV was detected in Fe-N-C, suggesting low residual Zn content.

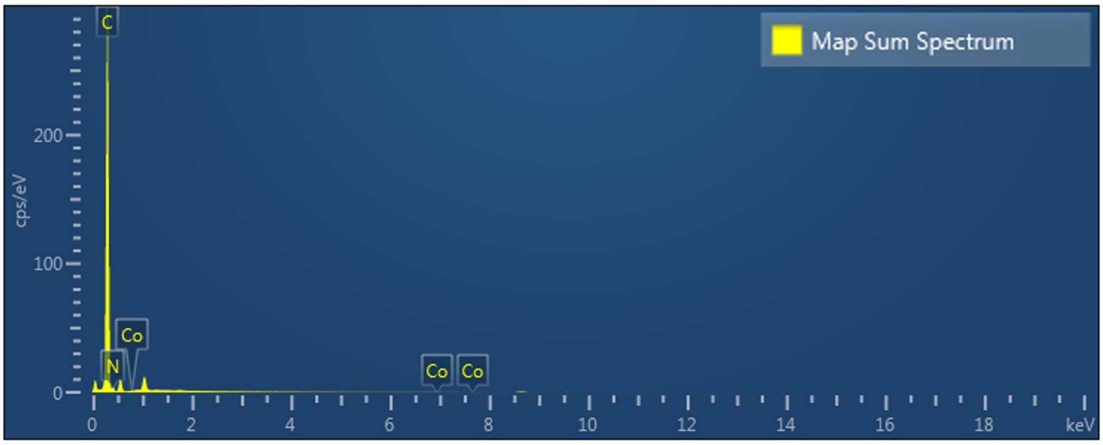

Figure S8. Energy dispersive spectroscopy (EDS) characterization of Co-N-C. No visible Zn signal locating at 8.63 keV was detected in Co-N-C, suggesting low residual Zn content.

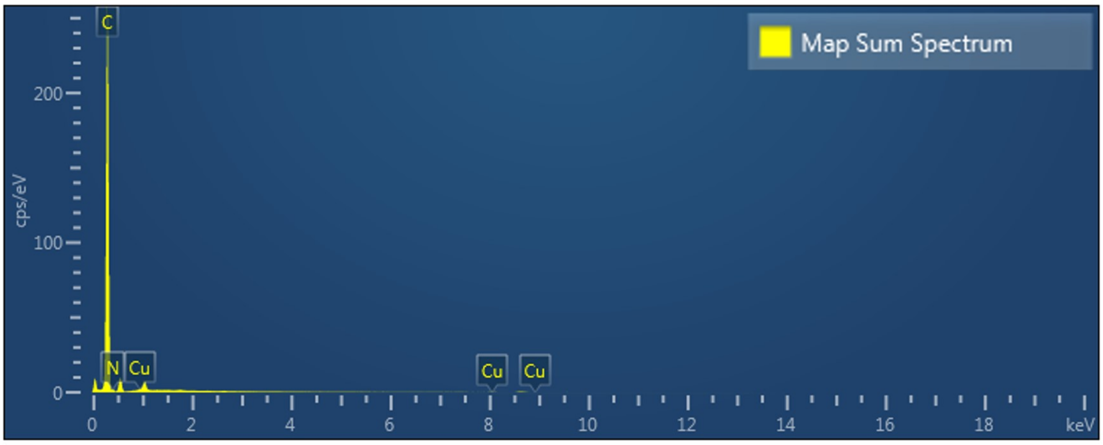

Figure S9. Energy dispersive spectroscopy (EDS) characterization of Cu-N-C. No visible Zn signal locating at 8.63 keV was detected in Cu-N-C, suggesting low residual Zn content.

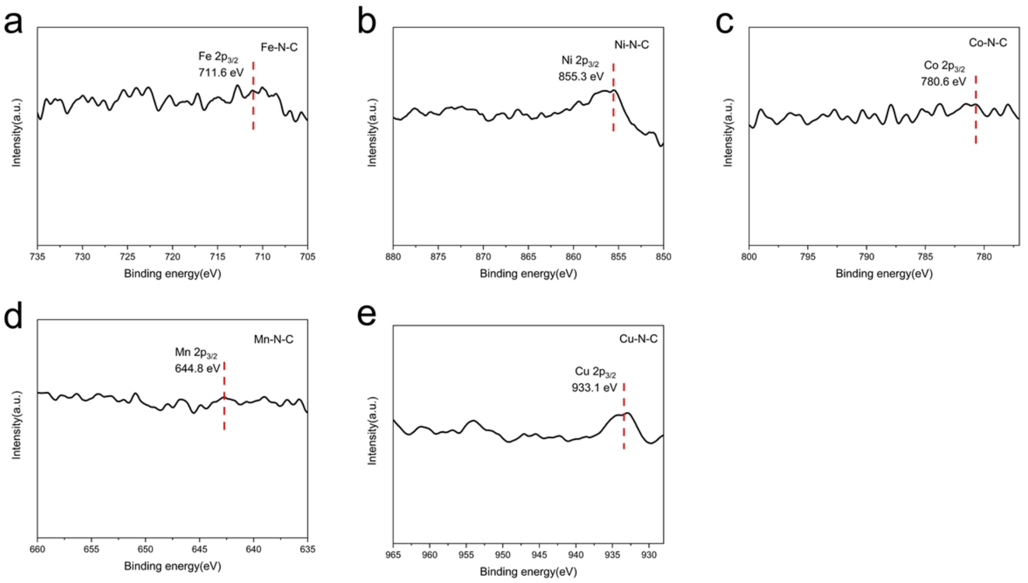

Figure S10. (a) Fe 2p spectrum of Fe-N-C, (b) Ni 2p spectrum of Ni-N-C, (c) Mn 2p spectrum of Mn-N-C, (d) Co 2p spectrum of Co-N-C and (e) Cu 2p spectrum of Cu-N-C

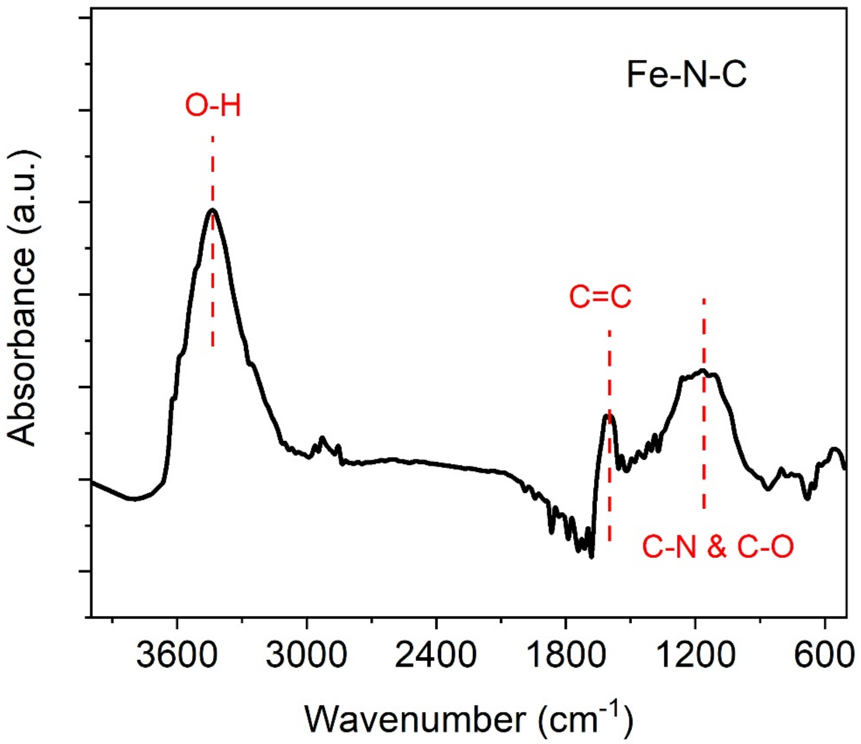

Figure S11. FT-IR spectra of Fe-N-C.

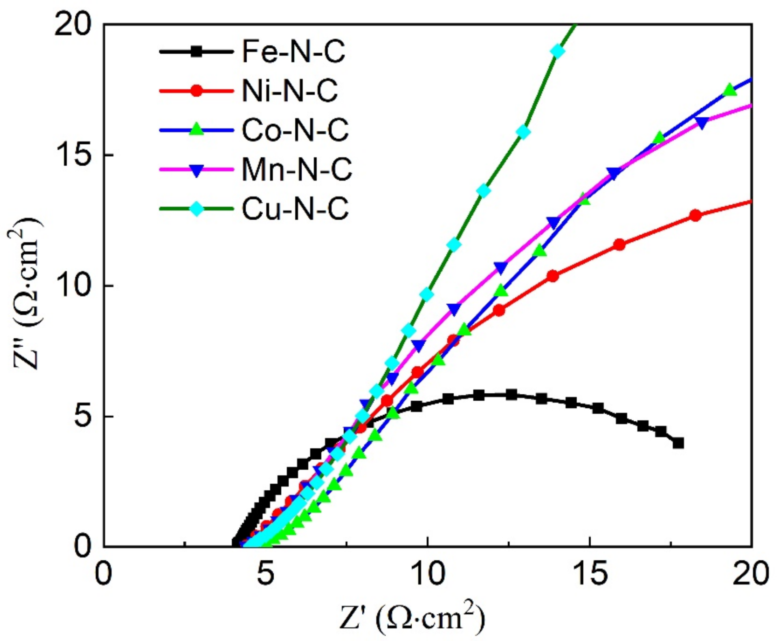

Figure S12. Nyquist plots of M-N-C catalysts in CO_2-saturated 0.5 M $KHCO_3$ solution at -0.5 V vs RHE.

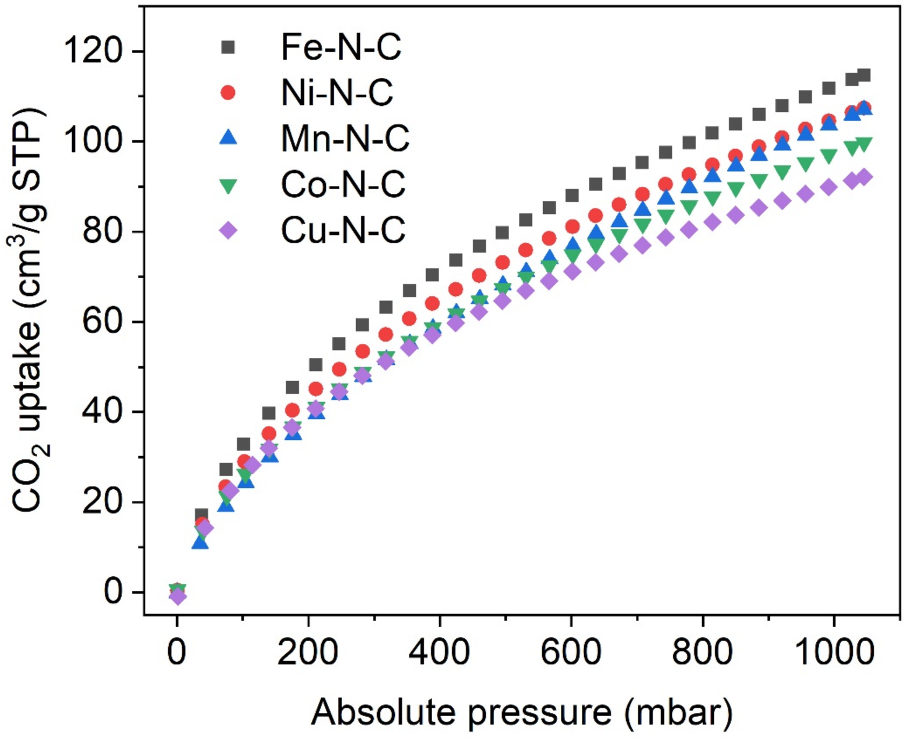

Figure S13. CO_2 physisorption isotherm at 273 K.

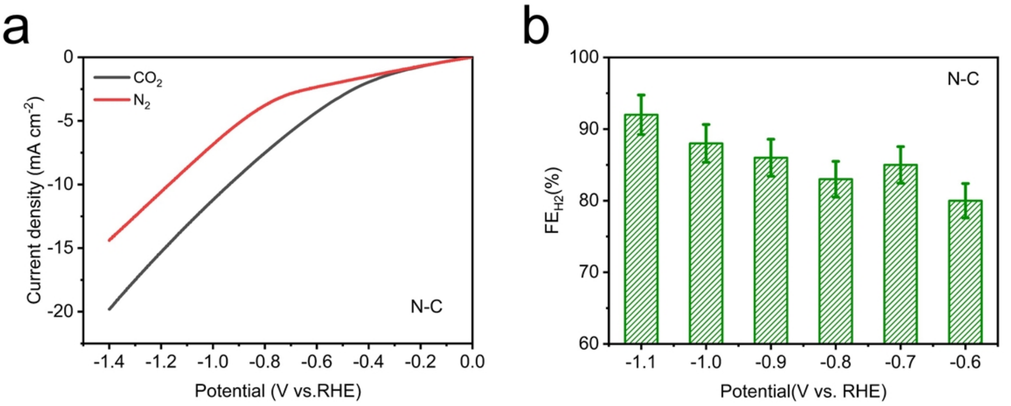

Figure S14. Electrocatalytic CO_2RR Performance of N-C catalyst using a flow cell (a) LSV curves in pure N_2- and CO_2-saturated 0.5 M $KHCO_3$ at a scan rate of 10 mV s^{-1}. (b) FE for H_2 production measured in CO_2-saturated 0.5 M $KHCO_3$

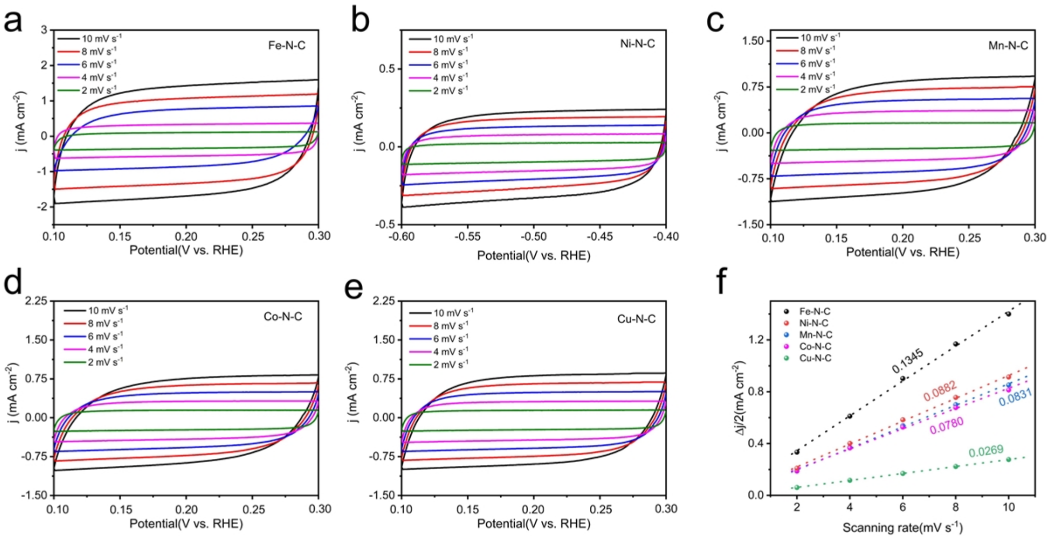

Figure S15. CV curves of (a) Fe-N-C, (b) Ni-N-C, (c) Mn-N-C, (d) Co-N-C and (e) Cu-N-C. The CV measurements were performed in CO_2-saturated 0.5 M $KHCO_3$ at various scan rates: 2, 4, 6, 8 and 10 mV s^{-1}. (e) A plot of changing current density against scan rates for electrochemically active surface area (ECSA) measurements. As clearly shown, ECSA of the M-N-C catalysts increased in the following order of Fe-N-C > 7 Ni-N-C > Mn-N-C > Co-N-C > Cu-N-C, coinciding with the FE_{CO} results.

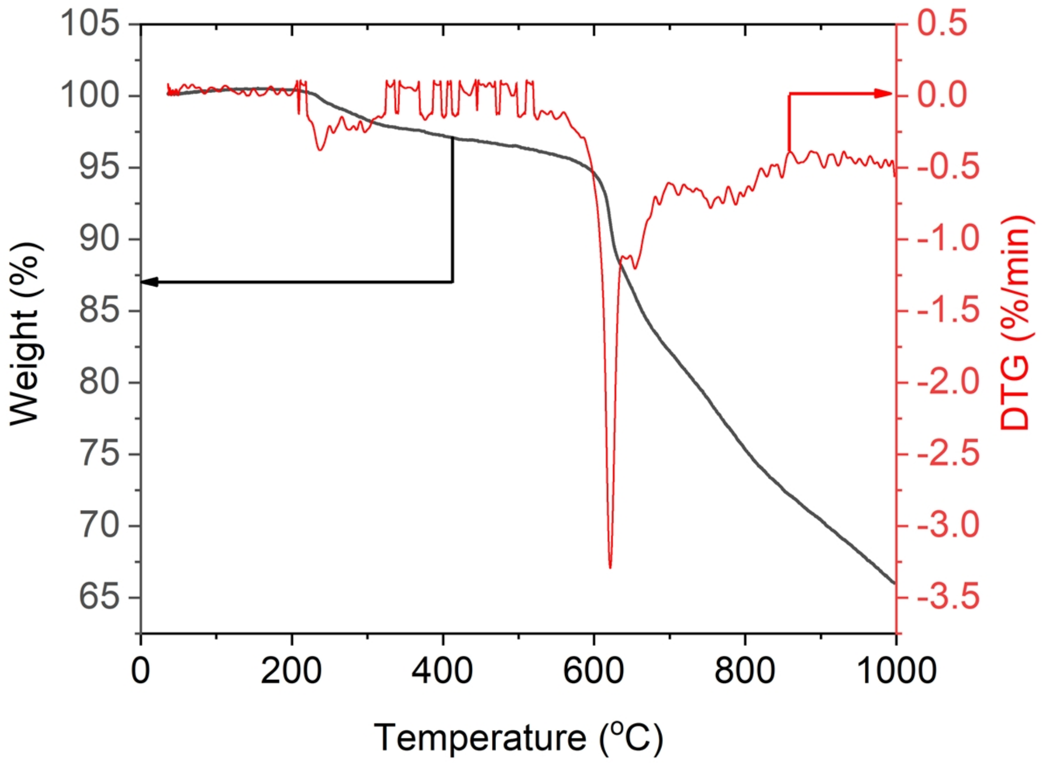

Figure S16. TG-DTG patterns of the composite of $Fe(NO_3)_3$ adsorbed within ZIF-8.

Table S1. Metal contents of all M-N-C samples determined by ICP-MS

Sample	Metal content (wt%)
Fe-N-C	0.48
Ni-N-C	0.65
Mn-N-C	0.58
Co-N-C	0.52
Cu-N-C	0.68
